# c-Myc overexpression sensitises colon cancer cells to camptothecin-induced apoptosis

**DOI:** 10.1038/sj.bjc.6601338

**Published:** 2003-10-28

**Authors:** D Arango, J M Mariadason, A J Wilson, W Yang, G A Corner, C Nicholas, M J Aranes, L H Augenlicht

**Affiliations:** 1 Albert Einstein Cancer Center, Montefiore Medical Center, Oncology Department, 111 East 210th St, Bronx, NY 10467, USA

**Keywords:** camptothecin, apoptosis, microarray, p21^Waf1/Cip1^

## Abstract

The proto-oncogene c-Myc is overexpressed in 70% of colorectal tumours and can modulate proliferation and apoptosis after cytotoxic insult. Using an isogenic cell system, we demonstrate that c-Myc overexpression in colon carcinoma LoVo cells resulted in sensitisation to camptothecin-induced apoptosis, thus identifying c-Myc as a potential marker predicting response of colorectal tumour cells to camptothecin. Both camptothecin exposure and c-Myc overexpression in LoVo cells resulted in elevation of p53 protein levels, suggesting a role of p53 in the c-Myc-imposed sensitisation to the apoptotic effects of camptothecin. This was confirmed by the ability of PFT-*α*, a specific inhibitor of p53, to attenuate camptothecin-induced apoptosis. p53 can induce the expression of p21^Waf1/Cip1^, an antiproliferative protein that can facilitate DNA repair and drug resistance. Importantly, although camptothecin treatment markedly increased p21^Waf1/Cip1^ levels in parental LoVo cells, this effect was abrogated in c-Myc-overexpressing derivatives. Targeted inactivation of p21^Waf1/Cip1^ in HCT116 colon cancer cells resulted in significantly increased levels of apoptosis following treatment with camptothecin, demonstrating the importance of p21^Waf1/Cip1^ in the response to this agent. Finally, cDNA microarray analysis was used to identify genes that are modulated in expression by c-Myc upregulation that could serve as additional markers predicting response to camptothecin. Thirty-four sequences were altered in expression over four-fold in two isogenic c-Myc-overexpressing clones compared to parental LoVo cells. Moreover, the expression of 10 of these genes was confirmed to be significantly correlated with response to camptothecin in a panel of 30 colorectal cancer cell lines.

The tumour-suppressor gene p53 plays a pivotal role in determining cell fate after cytotoxic insult with DNA-damaging agents. p53 is a transcription factor that can either trigger an apoptotic cell death by transcriptionally modulating multiple target genes, or promote cell cycle arrest, and thus facilitate DNA damage repair, through the upregulation of the cyclin-dependent kinase (cdk) inhibitor p21^Waf1/Cip1^ (el-Deiry *et al*, 1993; Miyashita *et al*, 1994). Recently, levels of the proto-oncogene c-Myc have been shown to be critical in switching the p53-dependent response from cell cycle arrest to apoptosis after gamma radiation or treatment with daunorubicin (Seoane *et al*, 2002), a topoisomerase II inhibitor that is not frequently used for the treatment of colorectal malignancies (Harvey *et al*, 1985). These effects are mediated through the ability of c-Myc to interact with Miz-1 and downregulate the expression of p21^Waf1/Cip1^, thus favouring the proapoptotic activities of p53. Previously, we demonstrated the clinical value of *c-myc* as a marker that predicts response to treatment with 5-fluorouracil (5FU), the standard chemotherapeutic agent used in the treatment of colorectal cancer (O'Dwyer and Stevenson, 1998). Low-level amplification of *c-myc*, together with a wild-type *p53* gene, identified a subset of patients with locally advanced colorectal cancer showing increased disease-free and overall survival in response to 5FU-based adjuvant therapy (Augenlicht *et al*, 1997; Arango *et al*, 2001).

Camptothecin is a topoisomerase I inhibitor that interferes with DNA replication and transcription by stabilising the covalent complex formed between Topoisomerase I and DNA. The camptothecin derivative CPT-11 (Irinotecan) has been shown to be a useful chemotherapeutic agent for the treatment of colorectal cancer patients, improving response rates and survival when used in combination with 5FU, and has also been shown to be effective in 5FU-resistant tumours (Cunningham *et al*, 1998; Douillard *et al*, 2000; Arango and Augenlicht, 2001). Recently, there has been significant progress in the identification of genetic markers that allow prediction of response to 5FU and/or oxaliplatin, which together with CPT-11 are the main chemotherapeutic agents used in the treatment of colorectal cancer (Augenlicht *et al*, 1997; Salonga *et al*, 2000; Arango *et al*, 2001, 2003; Park *et al*, 2001; Shirota *et al*, 2001). However, there is great need for markers predicting the efficacy of CPT-11 treatment. In this study, we hypothesised that the levels of c-Myc could modulate the cellular response to camptothecin. Using an *in vitro* isogenic system, we demonstrated the important role of c-Myc in the apoptotic response of colon cancer cells to camptothecin. Understanding of the molecular mechanisms underlying this observation would allow significant insight to be gained into the key determinants of response to chemotherapy and could identify new targets for intervention. Here we demonstrate a p53-dependent component in the c-Myc-imposed sensitisation to camptothecin-induced apoptosis. Moreover, forced expression of c-Myc resulted in reduced levels of p21^Waf1/Cip1^ despite elevated levels of p53, and targeted inactivation of p21^Waf1/Cip1^ resulted in increased sensitivity to apoptosis induced by this agent.

Finally, to identify additional markers capable of predicting apoptotic response to this agent, we used a cDNA microarray analysis approach. The levels of expression of 9216 sequences were assessed in camptothecin-resistant LoVo colon carcinoma cells and camptothecin-sensitive isogenic derivatives overexpressing c-Myc. Thiry-four sequences were identified exhibiting over 4-fold difference in expression. The potential of 10 of these genes as markers predicting response to camptothecin was confirmed by the significant correlation observed between the levels of expression of these genes and the extent of apoptosis induced by camptothecin in a panel of 30 different colorectal cancer cell lines.

## MATERIALS AND METHODS

### Cell lines and culture conditions

Colon adenocarcinoma LoVo cells and two isogenic clones overexpressing c-Myc (L2 and L3) have been extensively characterised and were maintained as described (Arango *et al*, 2001). TGR1 rat fibroblasts, isogenic HO15-19 cells with targeted disruption of both alleles of *c-myc*, and HOmyc3 cells in which c-Myc expression has been restored in the knockout cells (Mateyak *et al*, 1997) were kindly provided by Dr Sedivy (Brown University, RI, USA) and maintained with DMEM supplemented with 10% FBS and 1 × antibiotic/antimycotic (Invitrogen, Carlsbad, CA, USA). HCT116, a colon carcinoma cell line with a functional p53 gene, and an isogenic line with a targeted inactivation of p21^Waf1/Cip1^ (Bunz *et al*, 1998) were kind gifts of Dr Vogelstein (Johns Hopkins University School of Medicine). The panel of colorectal cancer cell lines used was: Caco-2, Colo201, Colo205, Colo320, DLD-1, HCT116, HCT-15, HCT-8, LoVo, LS174T, RKO, SK-CO-1, SW1116, SW403, SW48, SW480, SW620, SW837, SW948, T84 and WiDr (all from the American Type Culture Collection, Manassas, VA, USA), HT29, HT29-Cl.16E, HT29-Cl.19A (from Dr Laboisse, Institut National de la Sante et de la Recherche Medicale U539, France (Augeron and Laboisse, 1984)), LIM1215, LIM2405 (from Dr Whitehead, Vanderbilt University, Nashville, TN, USA (Whitehead *et al*, 1985; Devine *et al*, 1992)), HCC2998, KM12 (from the NCI-Frederick Cancer DCT tumour repository), RW2982 and RW7213 (Tibbetts *et al*, 1988). All the colorectal cancer cell lines were maintained in MEM supplemented with 10% FBS and 1 × antibiotic/antimycotic (Invitrogen, Carlsbad, CA, USA).

### Quantification of apoptosis

1 × 10^5^ cells were seeded in triplicate in six-well plates and 24 h later treated with the indicated concentrations of camptothecin (Calbiochem, La Jolla, CA, USA). In some experiments, Pifithrin-*α* (PFT-*α*; Calbiochem, La Jolla, CA, USA), a specific p53 inhibitor (Komarov *et al*, 1999), was also added at time 0 (15–30 *μ*M). The apoptotic response to camptothecin treatment was quantified after 72 h by propidium iodide (PI) staining and assessment of the proportion of cells with a subdiploid content of DNA by FACS analysis, as described (Arango *et al*, 2001).

### Western blot analysis

Both untreated and camptothecin-treated cultures (0.1 or 0.5 *μ*M for 24 h) growing in T75 flasks were rinsed twice with PBS, harvested and the pellet resuspended in 300 *μ*l of RIPA buffer (1% NP-40, 1% sodium deoxycholate, 0.1% SDS, 0.15 M NaCl, 0.01 M sodium phosphate pH 7.2, 2 mM EDTA, 50 mM sodium fluoride, 0.2 mM sodium vanadate and 100 U ml^−1^ aprotinin), lysed for 30 min on ice, and cleared by centrifugation. The appropriate volume of Laemmli loading buffer (6 ×) was added to 100 *μ*g aliquots and fractionated in 15% SDS–polyacrylamide gels. Proteins were transferred to a PVDF membrane (Amersham, Piscataway, NJ, USA), blocked with 10% nonfat milk for 1 h and then probed at room temperature with the appropriate primary antibody in 5% nonfat milk for 1 h. The following antibodies and dilutions were used: anti-p53 and anti-p21^Waf1/Cip1^ (DO-1, 1/7000 and H-164, 1/200, respectively; Santa Cruz Biotechnology, Santa Cruz, CA, USA), anti-*β*-actin (clone AC74, 1/1000; Sigma, Saint Louis, MO, USA). Membranes were washed three times with washing buffer (PBS with 0.1% Tween 20) and then probed with a peroxidase-conjugated secondary antibody for 1 h (Boehringer Mannheim, Indianapolis, IN, USA). After washing three times with washing buffer, the signal was detected using ECL plus (Amersham, Piscataway, NJ, USA) and a Storm PhosphorImager (Molecular Dynamics, Sunnyvale, CA, USA). The signal from the *β*-actin probe was used as a loading control.

### p21^Waf1/Cip1^ promoter activity

p21^Waf1/Cip1^ promoter activity was measured in parental LoVo cells and c-Myc-transfected L2 and L3 cells using a transient transfection assay. The p21P construct (Datto *et al*, 1995) has a 2.4 kb insert containing the p21^Waf1/Cip1^ promoter sequences in pGL2-basic (Promega, Madison, WI, USA), driving transcription of a firefly luciferase reporter gene. LoVo, L2 and L3 cells (5 × 10^4^ per well) were seeded in 24-well plates and cotransfected with p21P and TK-Renilla (Promega, Madison, WI, USA) 24 h later using GenePORTER II (Gene Therapy Systems, San Diego, CA, USA). After 48 h, cells were harvested, and firefly and Renilla luciferase activity levels assessed using the Dual Luciferase kit (Promega, Madison, WI, USA) according to the manufacturer's specifications. In addition, parental LoVo cells were cotransfected with both p21P and TK-Renilla as well as either p290-Myc(2,3), a c-Myc expression vector (Reed *et al*, 1989) or the empty p290 vector, to assess the effects of exogenous c-Myc on p21^Waf1/Cip1^ promoter activity. In all cases, values from TK-Renilla luciferase activity were used to correct for differences in transfection efficiency.

### Microarray analysis

Parental LoVo cells as well as L2 and L3 c-Myc transfectants (5 × 10^6^ cells) were seeded in T150 flasks, and harvested after 96 h. Total RNA was prepared using the RNeasy Midi kit (Qiagen, Valencia, CA, USA). RNA aliquots (100 *μ*g) were reverse-transcribed and labelled with Cy5 as described (Mariadason *et al*, 2000). All experimental samples were compared to a common reference RNA resulting from mixing equal amounts of total RNA from 12 different colon carcinoma cell lines. Reference RNA aliquots (100 *μ*g) were reverse-transcribed and labelled with Cy3 in parallel with the corresponding experimental samples, and then combined and hybridised to a 9216-sequence cDNA microarray from the Albert Einstein Cancer Center Microarray Facility, as described (Mariadason *et al*, 2000). Microarray slides were scanned and GenePix Pro 3.0 (Axon Instruments, Foster City, CA, USA) was used to quantify signal and background in the Cy5 and Cy3 channels for each spot, as well as the Cy5/Cy3 signal ratio and a normalisation factor that centred the average ratios over the slide on 1. These data were transferred to a Microsoft Excel spreadsheet, and normalised among arrays by multiplying the Cy5/Cy3 ratio by the normalisation factor, thereby allowing interarray comparison. All experiments were done in duplicate, beginning with different RNA preparations. Data from both hybridisations were averaged and used for subsequent analysis if there was a significant level of expression (defined as signal>background plus two standard deviations in the Cy5 and/or Cy3 channel). Relative expression between LoVo and either L2 or L3 cells was calculated by dividing the corresponding average values for each sequence. These values were entered into Microsoft Access and sequences with a difference in expression greater than four-fold between LoVo, and both L2 and L3 were identified. GeneCluster and TreeView software (Stanford University) were used to visualise the results.

### Quantitative real-time RT–PCR

The expression levels of 14 genes randomly selected from those identified as differentially expressed (over four-fold) in microarray experiments were confirmed using quantitative real-time RT–PCR. RNA aliquots (5 *μ*g) from the same preparations used for the microarray experiments were reverse transcribed using SuperScript II (Invitrogen, Carlsbad, CA, USA). PCR primers for specific target genes were designed using Primer Express software (Applied Biosystems, Foster City, CA, USA) and used to quantify the relative gene expression (see Table 1

**Table 1:** Genes differentially expressed in parental LoVo cells and c-Myc-overexpressing L2 and L3 isogenic cells

			**Microarray^a^**		**RT–PCR^b^**		**Correlation^c^**	
**Accession**	**Gene name**	**Function**	**L2/L**	**L3/L**	**L2/L**	**L3/L**	***r***	***P***
AA088861	Cadherin 17, LI cadherin (liver–intestine)	Cell adhesion	0.23	0.18	0.006	0.023	−0.16	
AA130579	Lectin, galactoside-binding, soluble, 4 (galectin 4)	Cell adhesion	7.14	10.89			0.33	
**N30868**	**Integrin, alpha 4**	**Cell adhesion**	**4.75**	**6.87**			0.48	**0.016**
**R60995**	**Coagulation factor C homologue, cochlin**	**Cell communication**	**0.12**	**0.17**			−0.54	**0.002**
AA453783	Mal, T-cell differentiation protein 2	Development–differentiation	6.26	4.5			0.35	
H17696	Myelin basic protein	Development–differentiation	12.03	10.41	0.260	0.350	−0.09	
**AA290737**	**Glutathione S-transferase M1**	**Drug metabolism/resistance**	**0.17**	**0.25**	**0.002**	**0.012**	−0.43	**0.019**
H29521	ATP-binding cassette 3	Drug metabolism/resistance	0.14	0.19	0.003	0.001	−0.09	
H88329	Calbindin 1, (28 kDa)	Drug metabolism/resistance	0.11	0.1	0.004	0.001	0.00	
**AA664101**	**Aldehyde dehydrogenase 1 family, member A1**	**Drug metabolism/resistance**	**44.09**	**28.97**	**832**	**1137**	0.49	**0.006**
AA031513	Matrix metalloproteinase 7 (matrilysin, uterine)	Extracellular matrix	20.68	17.23			−0.06	
AA056013	Microfibril-associated glycoprotein-2	Extracellular matrix	0.19	0.2			0.11	
AA479199	Nidogen 2	Extracellular matrix	0.21	0.22	0.004	0.002	−0.14	
AA458965	Natural killer cell transcript 4	Immune/inflammatory	0.08	0.16			−0.07	
AA682631	Calcineurin A alpha	Kinase/phosphatase	0.23	0.24			−0.28	
**AA431773**	**Fatty acid desaturase 1**	**Lipid metabolism**	**0.18**	**0.21**	**0.005**	**0.007**	−0.59	**0.001**
AA432066	Sarcoglycan, epsilon	Metabolism	0.23	0.2			−0.12	
AA434024	Lanosterol synthase	Metabolism	0.23	0.24	0.101	0.210	−0.14	
AA401137	Lipocalin 2 (oncogene 24p3)	Oncogene	10.98	8.89			0.12	
AA045436	Basic leucine zipper transcription factor MafG	Oncogene	5.07	4.33	1.370	4.820	−0.15	
**N63943**	**Lysozyme (renal amyloidosis)**	**Antimicrobial**	**10.01**	**8.18**	**2161.8**	**1817.5**	0.55	**0.002**
**AA134814**	**TRAF family member-associated NFKB activator**	**Signal transduction**	**4.83**	**4.44**			0.55	**0.013**
AA598567	Myelin gene expression factor 2	Transcriptional coactivator	5.02	4.85			−0.05	
AA055486	Tripartite motif-containing 29	Transcription factor	5.35	4.84			−0.10	
**AI017703**	**Eukaryotic translation initiation factor 3, subunit 3**	**Translation/AA biochemistry**	**5.11**	**4.25**			0.45	**0.013**
R38343	Protein tyrosine phosphatase, receptor type, G	Transmembrane receptor	0.23	0.18			−0.16	
T89391	Caveolin 2	Tumour suppressor	0.19	0.22	0.025	0.067	−0.01	
**AA112057**	**KIAA0143 protein**	**Unknown**	**6.73**	**5.57**			0.40	**0.031**
AA282134	OK/SW-cl.68 mRNA, complete cds	Unknown	16.06	10.51	774.2	568.4	0.12	
AA702350	Autism-related protein 1	Unknown	0.06	0.08			−0.04	
AA702949	KIAA0443 gene product	Unknown	0.11	0.16			−0.06	
**R40481**	**cDNA FLJ34699 fis, clone MESAN2002186**	**Unknown**	**6.57**	**4.55**			0.39	**0.037**
T62854	Hypothetical protein FLJ22662	Unknown	0.14	0.19			0.06	
W16832	Muscleblind-like protein MBLL39	Unknown	4.48	4.93	39.1	87.0	0.19	

Genes with significant correlations between their expression levels and apoptotic response to camptothecin in a panel of 30 colorectal cancer cell lines are shown in bold font.

a The relative mRNA levels are shown for the 34 genes with a difference in expression greater than four-fold in LoVo *vs* L2 and L3 in the microarray experiments (mean of two experiments).

b The relative expression levels are measured using quantitative real-time RT–PCR for 14 of these 34 genes.

c Pearson's correlation for gene expression and response to camptothecin in 30 colorectal cancer cell lines.

; primer sequences available at www.realtimeprimers.org). 10 ng cDNA aliquots from LoVo, L2 and L3 cells were amplified with specific primers using the SYBR green Core Reagents Kit and a 7900HT real-time PCR instrument (Applied Biosystems, Foster City, CA, USA). Expression of each gene was standardised using GAPDH as a reference, and relative levels in LoVo, L2 and L3 cells were quantified calculating 2^ΔΔ*C*_T_^, where ΔΔ*C*_T_ is the difference in *C*_T_ (cycle number at which the amount of amplified target reaches a fixed threshold) between target and reference, relative to parental LoVo cells.

## RESULTS

### Overexpression of c-Myc sensitises colon carcinoma cells to apoptosis induced by camptothecin

**Figure 1:**
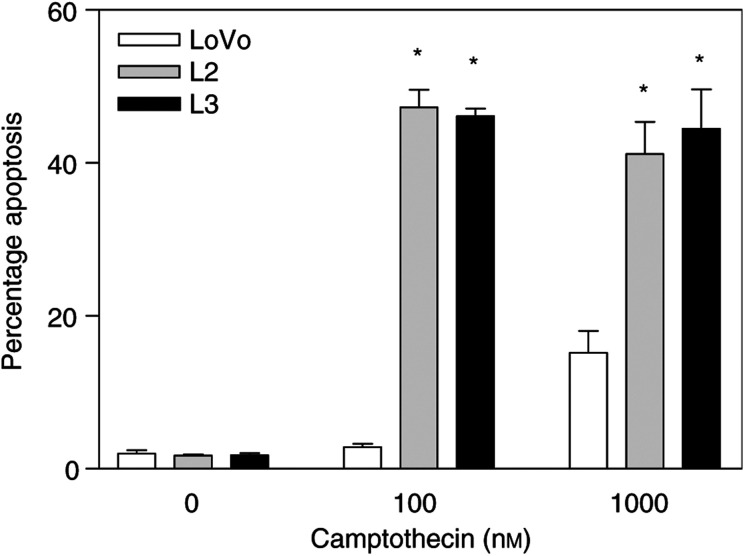
c-Myc overexpression sensitises colon cancer cells to camptothecin-induced apoptosis. The higher c-Myc levels and activity found in LoVo cells transfected with c-Myc (L2 and L3 cells) resulted in significant sensitisation to camptothecin-induced apoptosis, as demonstrated by PI staining and FACS analysis of cells exposed to 100 or 1000 nM camptothecin. The mean of three experiments±standard error of the mean is shown. Asterisks indicate significant differences (Student's *t*-test; *P*<0.0001) between L2 or L3 cells and parental LoVo cells.

To investigate the possible role of c-Myc in determining the apoptotic response of colon cancer cells to camptothecin, we used an isogenic *in vitro* system engineered by stably introducing a c-Myc expression vector into the LoVo colon cancer cell line (Arango *et al*, 2001). Two different derivative clones, L2 and L3, have been extensively characterised and shown to have c-Myc levels three- and eight-fold higher than parental LoVo cells, respectively, and correspondingly increased c-Myc transactivation activity levels (Arango *et al*, 2001). Quantification of the number of apoptotic cells after exposure to 100 or 1000 nM camptothecin for 72 h demonstrated that the higher c-Myc levels in L2 and L3 cells resulted in significantly (*P*<0.0001) increased apoptosis induced by this agent (Figure 1

).

**Figure 2:**
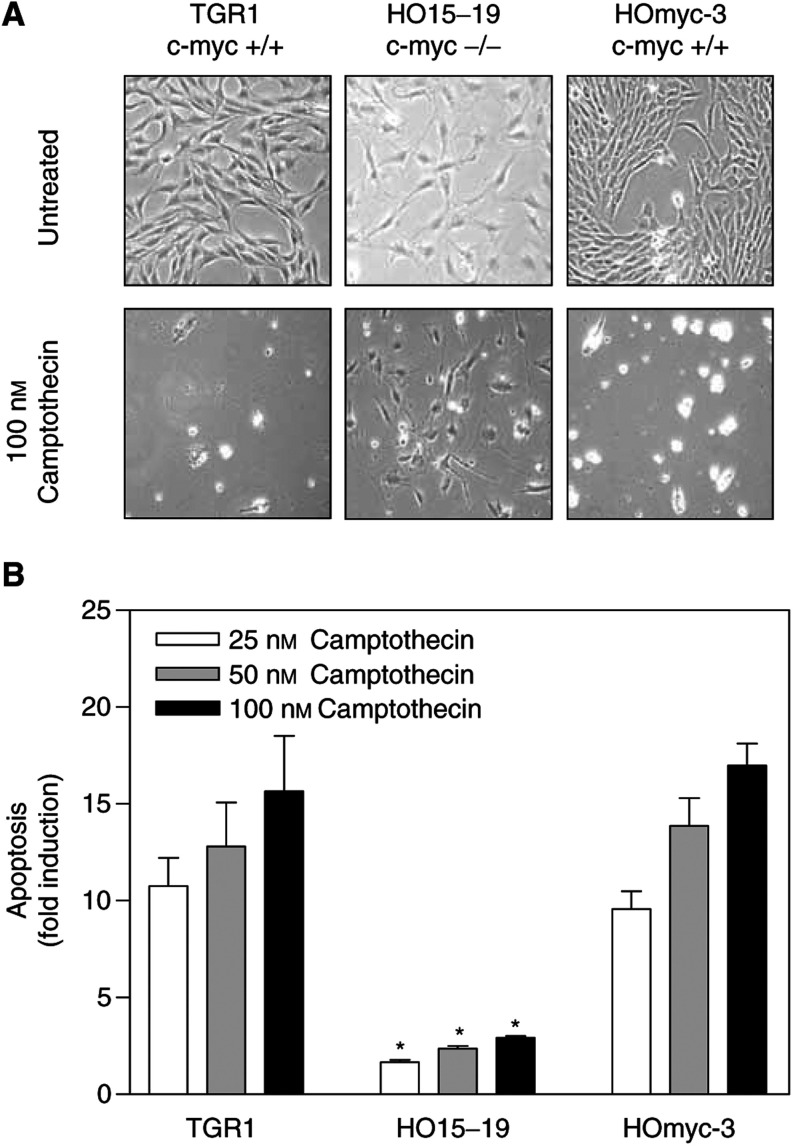
c-Myc levels modulate the apoptotic response to camptothecin in TGR1 cells. Panel (**A**) shows representative phase-contrast micrographs of TGR1 cells and derivatives exposed to 100 nM camptothecin for 72 h. In panel (**B**), the apoptotic response to camptothecin was quantified by PI staining and FACS analysis. Targeted deletion of the c-Myc gene in TGR1 cells (HO15-19 cells) results in a significant reduction in camptothecin-induced apoptosis (**A**, **B**). Restoring c-Myc expression in c-Myc-deficient HO15-19 cells (HOmyc-3 cells) results in a significant increase in apoptosis. The mean of three experiments±standard error of the mean is shown in panel (**B**). Asterisks indicate significant differences (Student's *t*-test; *P*<0.025) between c-Myc null HO15-19 cells and both parental TGR1 cells and c-Myc rescue HOmyc-3 cells for a given dose.

To further confirm the observed c-Myc-induced sensitisation to the apoptotic effects of camptothecin, we used TGR1 rat fibroblasts and their derivative, HO15-19 cells, which contain a homozygous deletion of the *c-myc* gene (Mateyak *et al*, 1997). In agreement with the results obtained using the LoVo system, the apoptotic response of TGR1 cells to camptothecin was significantly (*P*<0.025) reduced in c-Myc-deficient HO15-19 isogenic cells exposed for 72 h to concentrations of camptothecin ranging from 25 to 100 nM (Figure 2A, B

). Moreover, when c-Myc expression was rescued in HO15-19 cells (HOmyc3 cells), their response to camptothecin returned to that observed in parental TGR1 cells (Figure 2A, B). Collectively, these results demonstrate an important role for c-Myc in the apoptotic response to camptothecin.

### Role of p53 in c-Myc-imposed sensitisation to camptothecin-induced apoptosis

**Figure 3:**
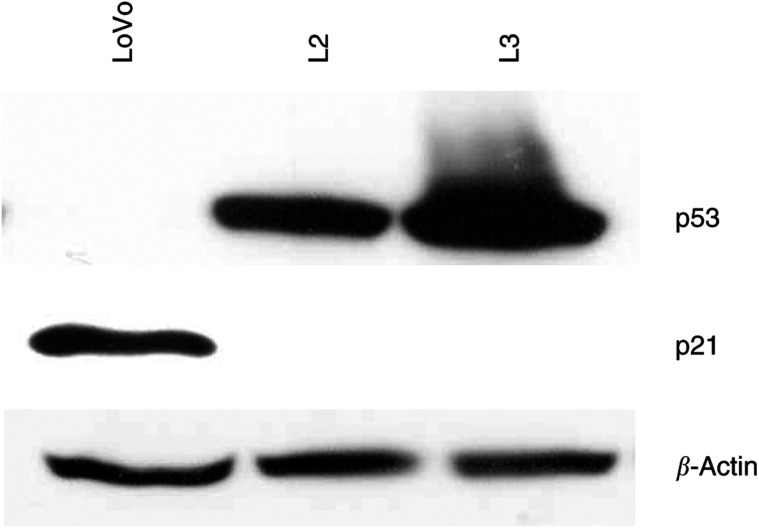
Effects of c-Myc overexpression on p53 and p21^Waf1/Cip1^ levels. Western blot analysis demonstrated that forced overexpression of c-Myc in LoVo cells (L2 and L3 cells) results in increased p53 and reduced p21^Waf1/Cip1^ protein levels.

**Figure 4:**
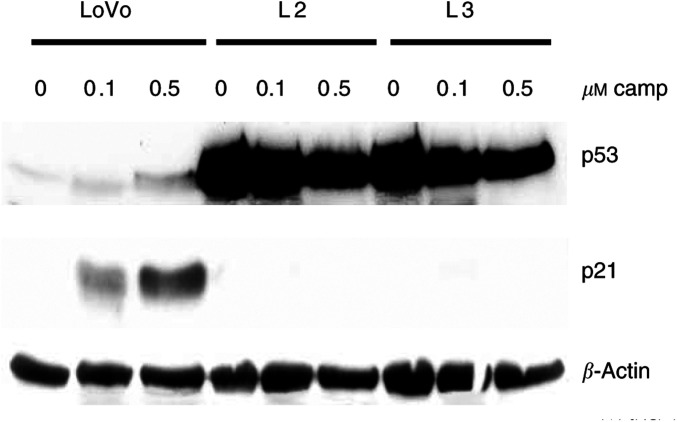
Effects of camptothecin treatment on p53 and p21^Waf1/Cip1^ levels. Western blot analysis demonstrated that exposure to 0.1–0.5 *μ*M camptothecin for 24 h results in a modest induction of p53 and significant accumulation of p21^Waf1/Cip1^ in parental LoVo cells. Overexpression of c-Myc in L2 and L3 cells completely abrogates upregulation of p21^Waf1/Cip1^ levels in response to camptothecin, despite the elevated p53 levels.

**Figure 5:**
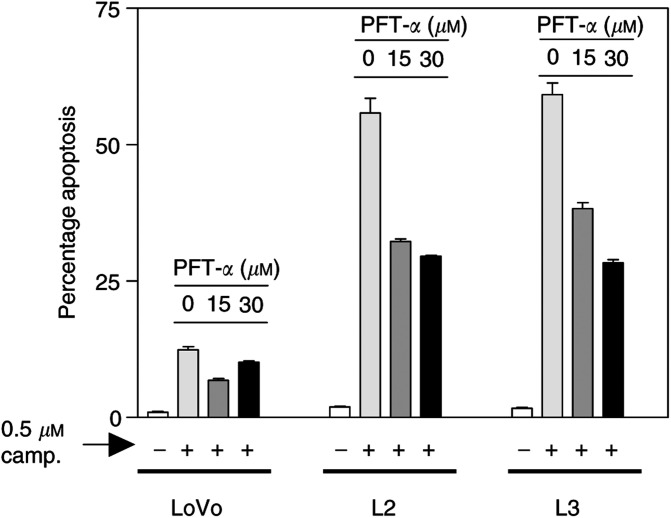
Role of p53 in the c-Myc imposed sensitisation to camptothecin-induced apoptosis. Exposure of L2 and L3 cells to camptothecin for 72 h in the presence of the specific inhibitor of p53 PFT-*α* demonstrated a p53-dependent component in their increased apoptotic response to this agent compared to parental LoVo cells. Mean of three experiments±standard error of the mean.

c-Myc has been shown to regulate p53 levels both directly and through modulation of p19^ARF^ (Reisman *et al*, 1993; Tavtigian *et al*, 1994; Zindy *et al*, 1998). In addition, p53 has been reported to modulate the cellular response to several chemotherapeutic agents, including camptothecin and its derivatives (Gupta *et al*, 1997; Yang *et al*, 1996). Therefore, to investigate the possible role of p53 in the increased apoptotic response to camptothecin imposed by c-Myc overexpression, we assessed the relative levels of this tumour suppressor in p53 wild-type LoVo cells and c-Myc-transfected clones L2 and L3. c-Myc overexpression in L2 and L3 cells resulted in markedly increased p53 protein levels (Figure 3

), consistent with the chemosensitive phenotype of these cells. Moreover, treatment of parental LoVo cells with 0.1 or 0.5 *μ*M camptothecin for 24 h lead to modestly increased p53 levels (Figure 4

). However, Figure 4 shows that p53 levels in camptothecin-treated parental LoVo cells remained significantly lower than in untreated L2 and L3 cells. To directly assess the functional role of p53 in camptothecin-induced apoptosis, c-Myc-overexpressing L2 and L3 cells were exposed to camptothecin in the presence of doses of PFT-*α*, a specific inhibitor of p53 (Komarov *et al*, 1999), that we have previously shown to inhibit 5FU-induced apoptosis in this system (Arango *et al*, 2001). Here, inhibition of p53 function resulted in over 50% reduction in the apoptotic response to 0.5 *μ*M camptothecin, demonstrating a p53-dependent component in the c-Myc-dependent increase in apoptosis following treatment with this agent (Figure 5

).

### Role of p21^Waf1/Cip1^ in c-Myc-imposed sensitisation to camptothecin-induced apoptosis

**Figure 6:**
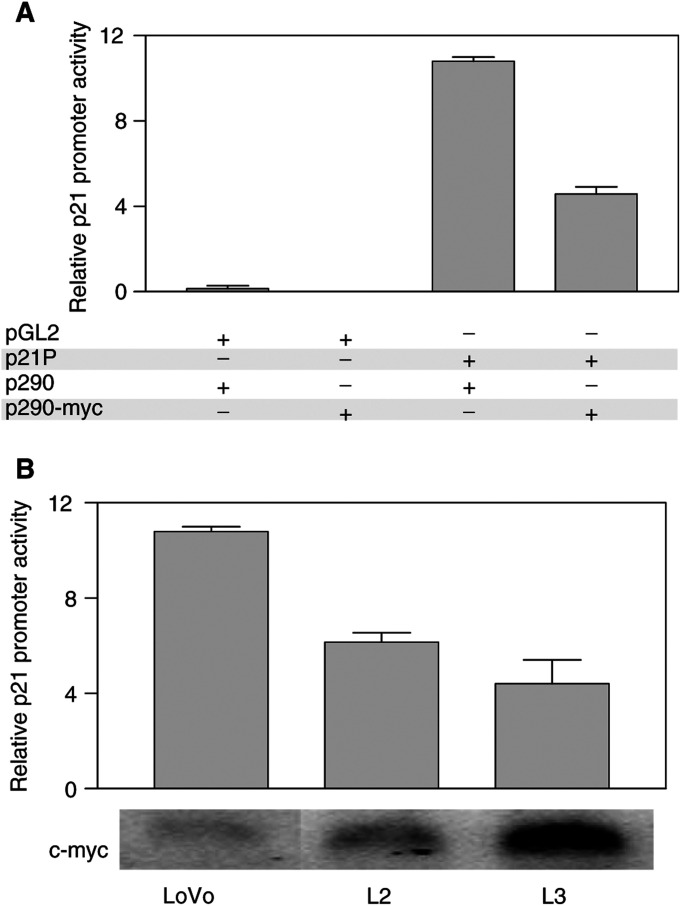
effects of c-Myc on p21^Waf1/Cip1^ promoter activity. (**A**) Parental LoVo cells were transfected with a vector containing the p21^Waf1/Cip1^ promoter sequences upstream of a Firefly Luciferase reporter gene (p21P) and a c-Myc expression vector (p290-Myc). This demonstrated a c-Myc-dependent reduction in p21^Waf1/Cip1^ promoter activity when compared to cells transfected with p21P and p290 empty vector (two-fold). Additional controls showed that transfection of LoVo cells with pGL2 (p21P without the p21^Waf1/Cip1^ promoter sequence) and either p290 or p290-Myc resulted in minimal luciferase activity. (**B**) LoVo, L2 and L3 cells were transfected with p21P to measure the differences in promoter activity. The increased c-Myc levels in L2 and L3 resulted in a c-Myc dose-dependent reduction of p21^Waf1/Cip1^ promoter activity. Relative c-Myc mRNA levels are shown underneath the histogram. Cotransfection with the plasmid TK-Renilla was used to correct for differences in transfection efficiency. The experiments were done three times in triplicate, and results of a representative experiment are shown.

p53 can strongly induce apoptosis by transcriptionally regulating the expression of a number of key players in the apoptotic cascade (Miyashita *et al*, 1994). However, p53 can alternatively induce cell cycle arrest and promote DNA damage repair through upregulation of the cdk inhibitor p21^Waf1/Cip1^ (el-Deiry *et al*, 1993). c-Myc overexpression in LoVo cells resulted in significantly increased p53 levels, which could lead to increased p21^Waf1/Cip1^ levels. However, c-Myc has been shown to directly downregulate p21^Waf1/Cip1^ levels (Mitchell and El-Deiry, 1999; Gartel *et al*, 2001). Therefore, we decided to assess the overall effects of c-Myc overexpression on the transcriptional activity of the p21^Waf1/Cip1^ promoter. First, we investigated whether c-Myc can reduce p21^Waf1/Cip1^ promoter activity in parental LoVo cells. Cotransfection of a c-Myc expression vector and a construct containing the p21^Waf1/Cip1^ promoter region upstream of a luciferase reporter gene (p21P plasmid), demonstrated that c-Myc could effectively reduce the promoter activity of p21^Waf1/Cip1^ in LoVo cells (Figure 6A

). Next, we compared p21^Waf1/Cip1^ promoter activity in parental LoVo cells and L2 and L3 cells showing elevated levels of c-Myc and p53. The promoter activity of p21^Waf1/Cip1^ was found to be significantly reduced in L2 and L3 cells compared to parental LoVo cells (1.75- and 2.5-fold downregulation, respectively; Figure 6B). Consistent with the reduced transcriptional activity of the p21^Waf1/Cip1^ promoter, Western blot analysis demonstrated that the higher c-Myc levels in LoVo transfectants (L2 and L3 cells) resulted in a significant reduction in p21^Waf1/Cip1^ protein (Figure 3), despite the higher p53 levels.

**Figure 7:**
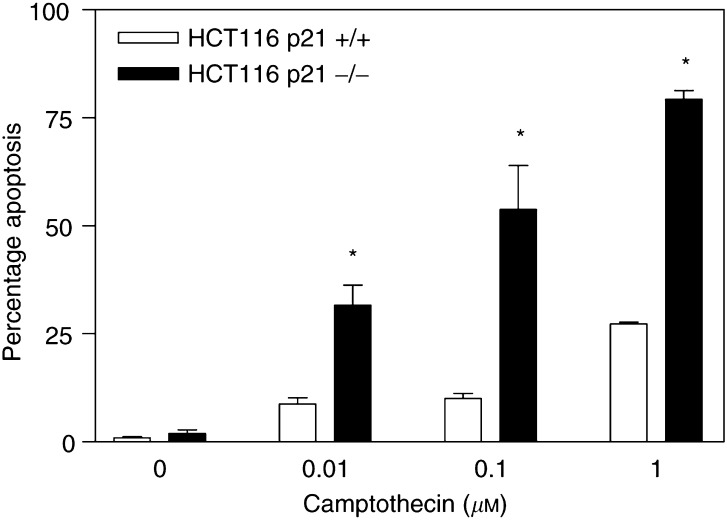
Role of p21 in the apoptotic response to camptothecin. Targeted inactivation of both alleles of p21^Waf1/Cip1^ in HCT116 colon cancer cells resulted in significant sensitisation to camptothecin-induced apoptosis. The mean of four different experiments±standard error of the mean is shown. Asterisks indicate significant differences (Student's *t*-test, *P*<0.004) between apoptotic levels in HCT116 p21+/+ and −/− for a given dose.

Importantly, although exposure of parental LoVo cells to 0.1 or 0.5 *μ*M camptothecin for 24 h resulted in upregulation of p53 and the p53 target gene p21^Waf1/Cip1^, c-Myc-overexpressing L2 and L3 cells failed to upregulate p21^Waf1/Cip1^ protein levels in response to camptothecin treatment (Figure 4), strongly suggesting that p21^Waf1/Cip1^ levels could modulate apoptosis induced by camptothecin. To directly investigate this possibility, we used an engineered *in vitro* system where both alleles of the p21^Waf1/Cip1^ gene have been inactivated by targeted deletion in HCT116 cells, a colon cancer cell line with a wild-type p53 gene (Bunz *et al*, 1998). Inactivation of p21^Waf1/Cip1^ in these cells resulted in significantly (*P*<0.004) increased sensitivity to apoptosis induced by camptothecin compared to parental wild-type p21^Waf1/Cip1^ HCT116 cells (Figure 7

), further demonstrating a role for p21^Waf1/Cip1^ in regulating response of colon cancer cells to camptothecin.

### Identification of additional markers predicting response to camptothecin

**Figure 8:**
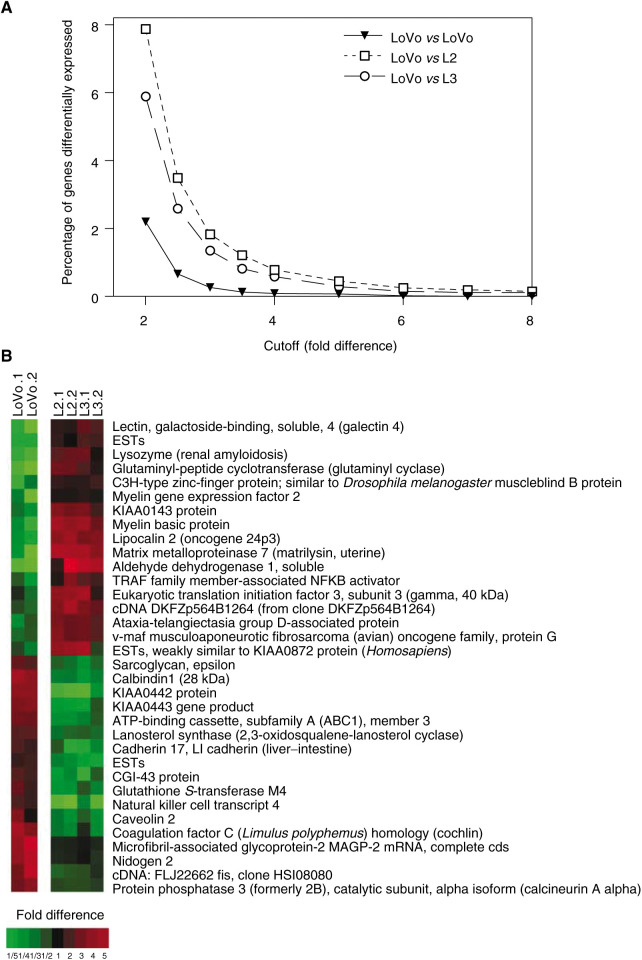
cDNA microarray analysis of LoVo and isogenic L2 and L3 cells overexpressing c-Myc. (**A**) The number of genes identified as differentially expressed is a function of the cutoff used. Selection of a stringent cutoff value of four-fold reduces the number of false positives expected when assessing differences in gene expression between LoVo and L2/L3 cells. (**B**) TreeView image showing the expression profile of the 34 genes with over four-fold expression difference between LoVo and L2/L3 that characterise camptothecin-resistant and -sensitive cells. Both replicas are shown. Red and green indicate genes that are over-represented and under-represented, respectively, relative to the reference RNA used (see Materials and Methods).

To identify additional genes that could serve as markers predicting apoptotic response to camptothecin, we measured the relative expression of 9216 sequences, in duplicate, in resistant LoVo cells, as well as in sensitive c-Myc-overexpressing L2 and L3 isogenic cells, using cDNA microarray analysis. The complete databases are available at http://sequence.aecom.yu.edu/b ioinf/Augenlicht/default.html. First, to assess the background variability due to methodological and biological factors, we averaged the values for the two LoVo replicas and compared them to the average of another two different replicates of LoVo cells, and quantified the number of genes identified as differentially expressed as a function of selected cutoff values (Figure 8A

). Using a conservative cutoff value of four-fold, there were six sequences that would be considered differentially expressed between the two pairs of LoVo replicas. However, using the same four-fold cutoff, LoVo *vs* L2 and LoVo *vs* L3 differed in the expression of 63 and 47 genes, respectively. Moreover, 34 of those genes were differentially expressed over four-fold in both L2 and L3 compared to parental LoVo cells (Figure 8B; Table 1).

Utilisation of a four-fold change in expression for gene selection greatly minimises the probability of false positives. This was confirmed by quantitative real-time RT–PCR analysis of the relative expression levels of 14 of the 34 genes identified as differentially expressed by the microarray analysis. Good qualitative agreement was observed in changes in the levels of expression assessed by microarray and real-time RT–PCR for 13 out of the 14 genes tested by the two methods (Table 1). However, as observed in other studies (Sgroi *et al*, 1999; Menssen and Hermeking, 2002; Reinhold *et al*, 2003), the magnitude of the differences in gene expression assessed by quantitative real-time RT-PCR was greater than those observed using a cDNA microarray approach (Table 1).

Although a subset of the 34 genes differentially expressed over four-fold in LoVo cells and c-Myc-overexpressing L2 and L3 cells is likely to play a role in their differential apoptotic response to camptothecin, elevated c-Myc levels have been shown to affect other phenotypic characteristics of colon cancer cells, including cell cycle progression and basic metabolism (Tikhonenko *et al*, 1996; Dang, 1999; Menssen and Hermeking, 2002). Therefore, the subset of genes that may be specific markers of response to camptothecin is not clear. To identify this subset of genes, we screened the expression of these 34 sequences in a separate microarray database containing the expression profile of 30 different colorectal cancer lines assessed using the same 9216-sequence cDNA chips (Mariadason *et al*, 2003; the full database is available at http://sequence.aecom.yu.edu/b ioinf/Augenlicht/default.html). In addition, we have determined the relative sensitivity of these cell lines to camptothecin-induced apoptosis (percent apoptosis after 72 h exposure to 1 *μ*M camptothecin; Mariadason *et al*, 2003). Of the 34 genes that varied in expression over four-fold in parental LoVo cells and c-Myc-overexpressing L2 and L3 cells, 10 showed a significant correlation (*R*>0.39; *P*<0.037) between expression levels and response to camptothecin in each of the 30 colorectal cancer cell lines in our panel (Table 1). As a control, we selected the 34 genes showing the least difference in expression between resistant LoVo cells and sensitive L2 and L3 cells. None of these genes showed a significant correlation between expression levels and apoptotic response to camptothecin in the panel of 30 colorectal cancer cell lines (not shown), highlighting the potential of the genes modulated by c-Myc as biomarkers of response to camptothecin.

## DISCUSSION

The proto-oncogene c-Myc is overexpressed in approximately 70% of colorectal tumours (Erisman *et al*, 1985). Deregulation and overexpression of c-Myc and other oncogenes, in addition to having proliferative effects, is frequently associated with an apoptosis-prone phenotype, thus opening the possibility of therapeutic intervention (Prendergast, 1999). This study demonstrates that upregulation of c-Myc levels and activity in colon cancer cells results in markedly increased sensitivity to apoptosis induced by camptothecin. Forced overexpression of c-myc in LoVo colon cancer cells, induced significant accumulation of p53, and the increased apoptotic response to camptothecin was at least partially dependent upon p53 function. However, p53 can induce cell cycle arrest and facilitate DNA damage repair through the transcriptional activation of the cdk inhibitor p21^Waf1/Cip1^. The factors determining a p53-dependent apoptotic or cytostatic response to cytotoxic insult remain unclear. Recently, elevated c-Myc levels have been identified as one such factor favouring a p53-dependent apoptotic response (Seoane *et al*, 2002). Here we show that despite the elevated p53 levels in c-Myc-overexpressing L2 and L3 cells compared to parental LoVo cells, c-Myc directly repressed the transcriptional activity of p21^Waf1/Cip1^ and resulted in reduced p21^Waf1/Cip1^ protein levels. Moreover, although camptothecin treatment resulted in p21^Waf1/Cip1^ accumulation in resistant LoVo cells, sensitive L2 and L3 cells failed to upregulate p21^Waf1/Cip1^ levels in the presence of camptothecin. The functional relevance of this finding was further demonstrated using an isogenic *in vitro* system in which both alleles of p21^Waf1/Cip1^ were inactivated by homologous recombination in HCT116 cells. Inactivation of p21^Waf1/Cip1^ in HCT116 cells resulted in significantly increased apoptosis following camptothecin treatment compared to p21^Waf1/Cip1^ wild-type parental HCT116 cells. Collectively, these studies identified the c-Myc-imposed reduction in p21^Waf1/Cip1^ levels as an important factor determining the increased sensitivity to camptothecin-induced apoptosis associated with elevated c-Myc levels.

Despite significant progress in the identification of markers predicting response to other chemotherapeutic agents commonly used in the treatment of colorectal malignancies, namely, 5FU and oxaliplatin (Augenlicht *et al*, 1997; Salonga *et al*, 2000; Arango *et al*, 2001, 2003; Park *et al*, 2001; Shirota *et al*, 2001), there is a great need for clinical predictors of response to camptothecin derivatives. The capability of predicting response to the chemotherapeutic agents available for the treatment of colorectal cancer would allow tailoring of treatment to individual patients, thus maximising the probability of optimal response to therapy. Here we identified c-Myc as a marker predicting response to camptothecin. Moreover, dissection of the molecular mechanisms responsible for the c-Myc-imposed sensitisation to camptothecin demonstrated the key role of p21^Waf1/Cip1^ in determining response of colon cancer cells to this agent, and pointed at this cdk inhibitor as an additional predictor of response.

To gain further insight into the differences between colon cancer cells that vary in their apoptotic response to camptothecin, and to identify additional markers that could help recognise tumours that vary in their response to this agent, we assessed the level of expression of 9216 sequences in resistant LoVo cells, and in sensitive L2 and L3 derivatives, using cDNA microarray analysis. This identified 34 genes that varied in expression over four-fold in parental LoVo cells and c-Myc-overexpressing L2 and L3 cells. To determine which of these 34 genes are effective predictors of the apoptotic response to camptothecin, we utilised a database containing the levels of expression of the same 9216 sequences in a panel of 30 different colorectal cancer cell lines using cDNA microarray analysis. In addition, we have also determined the relative sensitivity of these 30 cell lines to camptothecin. To identify those sequences associated with differences in sensitivity to camptothecin, we assessed whether each one of the 34 genes differentially expressed in resistant LoVo and sensitive L2/L3 cells showed a significant correlation between expression levels and camptothecin-induced apoptosis in the 30 cell lines in our panel. This identified 10 genes with significant expression/response correlations (*R*>0.39 and *P*<0.037; Table 1). This list of candidate genes capable of predicting response to camptothecin contains genes involved in drug metabolism/resistance, signal transduction, protein translation and general metabolism (see Table 1). At least some of these genes have been shown to vary in expression in colorectal tumours, including GST-M1, ALDH and lysozyme (Yuen *et al*, 1998; Saadat and Saadat, 2001), and could play a functional role in the cellular response to camptothecin and its derivatives. Therefore, in addition to their value as markers predicting response to camptothecin, each of these genes could represent a new target for therapeutic intervention.

## References

[bib1] Arango D, Augenlicht LH (2001) New approaches to colorectal cancer treatment. In Recent Research Developments in Cancer, Vol. 3 pp 385–395. Trivandrum: Transworld Research Network

[bib2] Arango D, Corner GA, Wadler S, Catalano PJ, Augenlicht LH (2001) c-myc/p53 interaction determines sensitivity of human colon carcinoma cells to 5-fluorouracil *in vitro* and *in vivo*. Cancer Res 61: 4910–4915 11406570

[bib3] Arango D, Wilson AJ, Mariadason JM, Corner GA, Arañes MJ, Nicholas C, Augenlicht LH (2003) Molecular mechanisms of action and prediction of response to oxaliplatin in colorectal cancer cells. (submitted) 10.1038/sj.bjc.6602215 PMC2409767 15545975

[bib4] Augenlicht LH, Wadler S, Corner G, Richards C, Ryan L, Multani AS, Pathak S, Benson A, Haller D, Heerdt BG (1997) Low-level c-myc amplification in human colonic carcinoma cell lines and tumors: a frequent, p53-independent mutation associated with improved outcome in a randomized multi-institutional trial. Cancer Res 57: 1769–1775 9135021

[bib5] Augeron C, Laboisse CL (1984) Emergence of permanently differentiated cell clones in a human colonic cancer cell line in culture after treatment with sodium butyrate. Cancer Res 44: 3961–3969 6744312

[bib6] Bunz F, Dutriaux A, Lengauer C, Waldman T, Zhou S, Brown JP, Sedivy JM, Kinzler KW, Vogelstein B (1998) Requirement for p53 and p21 to sustain G2 arrest after DNA damage. Science 282: 1497–1501 9822382 10.1126/science.282.5393.1497

[bib7] Cunningham D, Pyrhonen S, James RD, Punt CJA, Hickish TF, Heikkila R, Johannesen TB, Starkhammar H, Topham CA, Awad L (1998) Randomised trial of irinotecan plus supportive care versus supportive care alone after fluorouracil failure for patients with metastatic colorectal cancer. The Lancet 352: 1413–1418 10.1016/S0140-6736(98)02309-5 9807987

[bib8] Dang CV (1999) c-Myc target genes involved in cell growth, apoptosis, and metabolism. Mol Cell Biol 19: 1–11 9858526 10.1128/mcb.19.1.1 PMC83860

[bib9] Datto MB, Yu Y, Wang XF (1995) Functional analysis of the transforming growth factor beta responsive elements in the WAF1/Cip1/p21 promoter. J Biol Chem 270: 28623–28628 7499379 10.1074/jbc.270.48.28623

[bib10] Devine PL, Birrell GW, Whitehead RH, Harada H, Xing PX, McKenzie IF (1992) Expression of MUC1 and MUC2 mucins by human tumor cell lines. Tumour Biol 13: 268–277 1283926 10.1159/000217775

[bib11] Douillard JY, Cunningham D, Roth AD, Navarro M, James RD, Karasek P, Jandik P, Iveson T, Carmichael J, Alakl M, Gruia G, Awad L, Rougier P (2000) Irinotecan combined with fluorouracil compared with fluorouracil alone as first-line treatment for metastatic colorectal cancer: a multicentre randomised trial. Lancet 355: 1041–1047 10744089 10.1016/s0140-6736(00)02034-1

[bib12] el-Deiry WS, Tokino T, Velculescu VE, Levy DB, Parsons R, Trent JM, Lin D, Mercer WE, Kinzler KW, Vogelstein B (1993) WAF1, a potential mediator of p53 tumor suppression. Cell 75: 817–825 8242752 10.1016/0092-8674(93)90500-p

[bib13] Erisman MD, Rothberg PG, Diehl RE, Morse CC, Spandorfer JM, Astrin SM (1985) Deregulation of c-myc gene expression in human colon carcinoma is not accompanied by amplification or rearrangement of the gene. Mol Cell Biol 5: 1969–1976 3837853 10.1128/mcb.5.8.1969-1976.1985 PMC366914

[bib14] Gartel AL, Ye X, Goufman E, Shianov P, Hay N, Najmabadi F, Tyner AL (2001) Myc represses the p21(WAF1/CIP1) promoter and interacts with Sp1/Sp3. Proc Natl Acad Sci USA 98: 4510–4515 11274368 10.1073/pnas.081074898 PMC31865

[bib15] Gupta M, Fan S, Zhan Q, Kohn KW, O'Connor PM, Pommier Y (1997) Inactivation of p53 increases the cytotoxicity of camptothecin in human colon HCT116 and breast MCF-7 cancer cells. Clin Cancer Res 3: 1653–1660 9815856

[bib16] Harvey J, Bonnem E, Grady K, Goodman A, Schein P (1985) Phase II study of daunorubicin in previously untreated patients with advanced colorectal carcinoma. Med Pediatr Oncol 13: 30–31 3969059 10.1002/mpo.2950130107

[bib17] Komarov PG, Komarova EA, Kondratov RV, Christov-Tselkov K, Coon JS, Chernov MV, Gudkov AV (1999) A chemical inhibitor of p53 that protects mice from the side effects of cancer therapy. Science 285: 1733–1737 10481009 10.1126/science.285.5434.1733

[bib18] Mariadason JM, Arango D, Shi Q, Wilson AJ, Corner GA, Nicholas C, Aranes MJ, Schwartz EL, Lesser M (2003) Gene expression profiling based prediction of response of colon carcinoma cells to chemotherapeutic agents. Cancer Res (in press) 14695196

[bib19] Mariadason JM, Corner GA, Augenlicht LH (2000) Genetic reprogramming in pathways of colonic cell maturation induced by short chain fatty acids: comparison with trichostatin A, sulindac, and curcumin and implications for chemoprevention of colon cancer. Cancer Res 60: 4561–4572 10969808

[bib20] Mateyak MK, Obaya AJ, Adachi S, Sedivy JM (1997) Phenotypes of c-Myc-deficient rat fibroblasts isolated by targeted homologous recombination. Cell Growth Differ 8: 1039–1048 9342182

[bib21] Menssen A, Hermeking H (2002) Characterization of the c-MYC-regulated transcriptome by SAGE: identification and analysis of c-MYC target genes. Proc Natl Acad Sci USA 99: 6274–6279 11983916 10.1073/pnas.082005599 PMC122939

[bib22] Mitchell KO, El-Deiry WS (1999) Overexpression of c-Myc inhibits p21WAF1/CIP1 expression and induces S-phase entry in12-O-tetradecanoylphorbol-13-acetate (TPA)-sensitive human cancer cells. Cell Growth Differ 10: 223–230 10319992

[bib23] Miyashita T, Krajewski S, Krajewska M, Wang HG, Lin HK, Liebermann DA, Hoffman B, Reed JC (1994) Tumor suppressor p53 is a regulator of bcl-2 and bax gene expression *in vitro* and *in vivo*. Oncogene 9: 1799–1805 8183579

[bib24] O'Dwyer PJ, Stevenson JP (1998) Chemotherapy of advanced colorectal cancer. In Gastrointestinal Oncology, Cancer Treatment and Research, Benson AL (ed) pp 111–152. Norwell: Kluwer Academic Publishers 10.1007/978-1-4615-4977-2_5 10326667

[bib25] Park DJ, Stoehlmacher J, Zhang W, Tsao-Wei DD, Groshen S, Lenz HJ (2001) A *Xeroderma pigmentosum* group D gene polymorphism predicts clinical outcome to platinum-based chemotherapy in patients with advanced colorectal cancer. Cancer Res 61: 8654–8658 11751380

[bib26] Prendergast GC (1999) Mechanisms of apoptosis by c-Myc. Oncogene 18: 2967–2987 10378693 10.1038/sj.onc.1202727

[bib27] Reed JC, Haldar S, Cuddy MP, Croce C, Makover D (1989) Deregulated BCL2 expression enhances growth of a human B cell line. Oncogene 4: 1123–1127 2789359

[bib28] Reinhold WC, Kouros-Mehr H, Kohn KW, Maunakea AK, Lababidi S, Roschke A, Stover K, Alexander J, Pantazis P, Miller L, Liu E, Kirsch IR, Urasaki Y, Pommier Y, Weinstein JN (2003) Apoptotic susceptibility of cancer cells selected for camptothecin resistance: gene expression profiling, functional analysis, and molecular interaction mapping. Cancer Res 63: 1000–1011 12615715

[bib29] Reisman D, Elkind NB, Roy B, Beamon J, Rotter V (1993) c-Myc trans-activates the p53 promoter through a required downstream CACGTG motif. Cell Growth Differ 4: 57–65 8494784

[bib30] Saadat I, Saadat M (2001) Glutathione S-transferase M1 and T1 null genotypes and the risk of gastric and colorectal cancers. Cancer Lett 169: 21–26 11410321 10.1016/s0304-3835(01)00550-x

[bib31] Salonga D, Danenberg KD, Johnson M, Metzger R, Groshen S, Tsao-Wei DD, Lenz HJ, Leichman CG, Leichman L, Diasio RB, Danenberg PV (2000) Colorectal tumors responding to 5-fluorouracil have low gene expression levels of dihydropyrimidine dehydrogenase, thymidylate synthase, and thymidine phosphorylase. Clin Cancer Res 6: 1322–1327 10778957

[bib32] Seoane J, Le HV, Massague J (2002) Myc suppression of the p21(Cip1) Cdk inhibitor influences the outcome of the p53 response to DNA damage. Nature 419: 729–734 12384701 10.1038/nature01119

[bib33] Sgroi DC, Teng S, Robinson G, LeVangie R, Hudson Jr JR, Elkahloun AG (1999) *In vivo* gene expression profile analysis of human breast cancer progression. Cancer Res 59: 5656–5661 10582678

[bib34] Shirota Y, Stoehlmacher J, Brabender J, Xiong YP, Uetake H, Danenberg KD, Groshen S, Tsao-Wei DD, Danenberg PV, Lenz HJ (2001) ERCC1 and thymidylate synthase mRNA levels predict survival for colorectal cancer patients receiving combination oxaliplatin and fluorouracil chemotherapy. J Clin Oncol 19: 4298–4304 11731512 10.1200/JCO.2001.19.23.4298

[bib35] Tavtigian SV, Zabludoff SD, Wold BJ (1994) Cloning of mid-G1 serum response genes and identification of a subset regulated by conditional myc expression. Mol Biol Cell 5: 375–388 8049528 10.1091/mbc.5.3.375 PMC301044

[bib36] Tibbetts LM, Chu MY, Vezeridis MP, Miller PG, Tibbetts LL, Poisson MH, Camara PD, Calabresi P (1988) Cell culture of the mucinous variant of human colorectal carcinoma. Cancer Res 48: 3751–3759 2837323

[bib37] Tikhonenko AT, Black DJ, Linial ML (1996) Viral Myc oncoproteins in infected fibroblasts down-modulate thrombospondin-1, a possible tumor suppressor gene. J Biol Chem 271: 30741–30747 8940053 10.1074/jbc.271.48.30741

[bib38] Whitehead RH, Macrae FA, St John DJ, Ma J (1985) A colon cancer cell line (LIM1215) derived from a patient with inherited nonpolyposis colorectal cancer. J Natl Cancer Inst 74: 759–765 3857372

[bib39] Yang B, Eshleman JR, Berger NA, Markowitz SD (1996) Wild-type p53 protein potentiates cytotoxicity of therapeutic agents in human colon cancer cells. Clin Cancer Res 2: 1649–1657 9816112

[bib40] Yuen ST, Wong MP, Chung LP, Chan SY, Cheung N, Ho J, Leung SY (1998) Up-regulation of lysozyme production in colonic adenomas and adenocarcinomas. Histopathology 32: 126–132 9543668 10.1046/j.1365-2559.1998.00339.x

[bib41] Zindy F, Eischen CM, Randle DH, Kamijo T, Cleveland JL, Sherr CJ, Roussel MF (1998) Myc signaling via the ARF tumor suppressor regulates p53-dependent apoptosis and immortalization. Genes Dev 12: 2424–2433 9694806 10.1101/gad.12.15.2424 PMC317045

